# Emerging Two-Dimensional Materials: Inspiring Nanotechnologies for Smart Energy Management

**DOI:** 10.3390/nano13081353

**Published:** 2023-04-13

**Authors:** Emiliano Bonera, Alessandro Molle

**Affiliations:** 1 Dipartimento di Scienza dei Materiali, Università di Milano-Bicocca, Via Cozzi 55, I-20125 Milano, Italy; 2 Consiglio Nazionale delle Ricerche (CNR), Istituto per la Microelettronica e Microsistemi (IMM), Unit of Agrate Brianza, Via C. Olivetti 2, I-20864 Agrate Brianza, Italy

Two-dimensional (2D) materials are a class of materials that can be reduced to a thickness of a few layers, exhibiting peculiar and innovative properties relative to their three-dimensional solid counterparts. These materials are characterized by unique and often tunable electronic, optical, mechanical, and chemical properties, which make them attractive for a wide range of applications, including electronics, optoelectronics, and sensing. In nanotechnology, there is a common thinking that this class could inherit the role historically assigned to traditional semiconductors and insulators, thus moving the bar of technological evolution further.

These materials are also gaining growing interest for their potential for energy-saving technologies, which include harvesting, conversion, and storage. This Special Issue collected contributions with respect to the modeling, synthesis, and characterization of two-dimensional materials, with special attention focused on their possible exploitation for energy-related applications. The editorial choice was to focus mainly on metal dichalcogenides [1,2], X-enes [3], and related materials, alongside their hetero-integration, functionalization, and engineering. Let us summarize quickly the results gathered.

At the beginning of a path of creation and the optimization of a new material or a new device, we can often find a model or a theoretical prediction. In this Special Issue, a theoretical study demonstrated the optimization of the atomic structure of several polymorphs of melem-based carbon nitrides for applications in photocatalysis [4]. Another theoretical study investigated in detail the electronic and optical properties in some allotropes of tellurium, a material with a promising perspective for photovoltaics and energy storage [5].

Regarding materials design and engineering, attention was focused on the deposition of 2D materials with energy management potential. In particular, we collected important contributions about the MoS2 deposition by means of chemical vapor deposition [6,7].

For the experimental investigation of the electrochemical properties of materials, we can read a meticulous in-depth analysis of the novel Pt3Te4 material in the form of nanostructured mitrofanovite [8], and we can also read a report on a detailed investigation of the behavior of lithium at bilayer-patched graphene on SiC [9]. Finally, for the thermal management of devices, a contribution was devoted to the measurement of the interface thermal resistance of black phosphorus on top of a gold substrate using Raman spectroscopy [10].

After the completion of the contribution, the time has come; indeed, it has been very pleasant for us, and at the end of this effort, we wish to thank all authors of this Special Issue for their outstanding and diverse interventions. We wish as well as to thank the referees for their patience and constructive interactions that led to a substantial improvement in the final form of the studies. Finally, we would also like to acknowledge the valuable and prompt support from Greta Zhang and the Editorial Office of *Nanomaterials* for allowing us to achieve this important collection of exciting novel scientific advancements.

## References

[1] Manzeli S., Ovchinnikov D., Pasquier D., Yazyev O.V., Kis A. (2017). 2D Transition Metal Dichalcogenides. Nat. Rev. Mater..

[2] Pi L., Li L., Liu K., Zhang Q., Li H., Zhai T. (2019). Recent Progress on 2D Noble-Transition-Metal Dichalcogenides. Adv. Funct. Mater..

[3] Grazianetti C., Martella C., Molle A. (2020). The Xenes Generations: A Taxonomy of Epitaxial Single-Element 2D Materials. Phys. Status Solidi (RRL)—Rapid Res. Lett..

[4] Ugolotti A., Di Valentin C. (2021). Ab-Initio Spectroscopic Characterization of Melem-Based Graphitic Carbon Nitride Polymorphs. Nanomaterials.

[5] Grillo S., Pulci O., Marri I. (2022). Evolution of the Electronic and Optical Properties of Meta-Stable Allotropic Forms of 2D Tellurium for Increasing Number of Layers. Nanomaterials.

[6] Tummala P.P., Martella C., Molle A., Lamperti A. (2022). Ambient Pressure Chemical Vapor Deposition of Flat and Vertically Aligned MoS2 Nanosheets. Nanomaterials.

[7] Martella C., Campi D., Tummala P.P., Kozma E., Targa P., Codegoni D., Bernasconi M., Lamperti A., Molle A. (2022). Extreme Bendability of Atomically Thin MoS2 Grown by Chemical Vapor Deposition Assisted by Perylene-Based Promoter. Nanomaterials.

[8] D’Olimpio G., Zhang L., Kuo C.-N., Farias D., Ottaviano L., Lue C.S., Fujii J., Vobornik I., Agarwal A., Torelli P. (2022). Efficient Hydrogen Evolution Reaction with Bulk and Nanostructured Mitrofanovite Pt3Te4. Nanomaterials.

[9] Shtepliuk I., Vagin M., Khan Z., Zakharov A.A., Iakimov T., Giannazzo F., Ivanov I.G., Yakimova R. (2022). Understanding of the Electrochemical Behavior of Lithium at Bilayer-Patched Epitaxial Graphene/4H-SiC. Nanomaterials.

[10] Bonera E., Molle A. (2022). Optothermal Raman Spectroscopy of Black Phosphorus on a Gold Substrate. Nanomaterials.

